# What, how, and why? – anti-EHEC phages and their application potential in medicine and food industry

**DOI:** 10.1007/s13353-024-00918-4

**Published:** 2024-11-11

**Authors:** Agnieszka Necel, Aleksandra Dydecka, Gracja Topka-Bielecka, Wojciech Wesołowski, Natalia Lewandowska, Sylwia Bloch, Bożena Nejman-Faleńczyk

**Affiliations:** 1 Department of Medical Microbiology, Faculty of Medicine, Medical University of Gdańsk, Dębowa 25, 80-204 Gdansk, Poland; 2 Univentum Labs Ltd., Aleja Grunwaldzka 472b, 80-309 Gdańsk, Poland; 3 Department of Molecular Biology, Faculty of Biology, University of Gdańsk, Wita Stwosza 59, 80-308 Gdansk, Poland; 4 BNF – New Bio Force sp. z o.o., Kartuska 420a, 80-125 Gdańsk, Poland

**Keywords:** EHEC, Bacteriophages, Food, Phage therapy

## Abstract

Enterohemorrhagic *Escherichia* *coli* (EHEC) are pathogens that, only in the United States, cause more than 250,000 foodborne infections a year. Since antibiotics or other antidiarrheal agents may increase the hemolytic-uremic syndrome (HUS) development risk, currently only supportive therapy, including hydration, is used. Therefore, many methods to fight EHEC bacteria focus on their use in food processing to prevent human infection. One of the proposed anti-EHEC agents is bacteriophages, known for their bactericidal effect, host specificity, and lack of cross-resistance with antibiotics. In this review article, we provide an overview of the characteristics like source of isolation, morphology, kinetics of life cycle, and treatment potential of over 130 bacteriophages able to infect EHEC strains. Based on the reviewed literature, we conclude that bacteriophages may play a highly significant role in regulating EHEC propagation. In addition, we also point out the phage features that should be taken into account not only when using bacteriophages but also when examining their properties. This may contribute to accelerating the pace of work on the preventive use of bacteriophages, which is extremely needed in the modern world of the food industry, but also stimulate interest in phages and accelerate regulatory work that would enable the use of bacteriophages also in medicine, to fight the drug-resistant strains.

## Introduction

Nature is a limitless source of bacteriophages as it is well known that they are the most abundant biological entities on Earth. The number of phages is estimated at 10^31^ in total, 10 times more than the number of bacterial cells (Weitz et al. 2013). Currently, the diversity of phages appears extremely high and within different environments, one can identify a wide range of phages exhibiting distinct morphologies and host ranges (Kaczorowska et al. 2019). The increasing tempo of scientific, medical, and industrial interest in phages perfectly shows that they are one of the main candidates for alternative or as a support to antibiotic therapy (Tagliaferri et al. 2019; Gu Liu et al. 2020). Such antimicrobials are extremely needed, especially in the current multi-drug resistance (MDR) era. However, the uselessness of antibiotics might not only come from the bacteria resistance. In the case of enterohemorrhagic *Escherichia coli* (EHEC), among which the MDR strains occur frequently, treatment with antibiotics may lead to exacerbation of the disease symptoms and even death (Kakoullis et al. 2019; Rehman et al. 2021). Therefore, research on the use of bacteriophages seems to be an appropriate direction for creating new strategies against these pathogens. In our review, we decided to focus on the characteristics of bacteriophages able to infect EHEC. In addition to host range, genomic characteristics, or cytotoxic effects, we also listed and discussed information on the phages' applicability, especially in the food industry.

## Sources and availability of phages

**Fig. 1:**
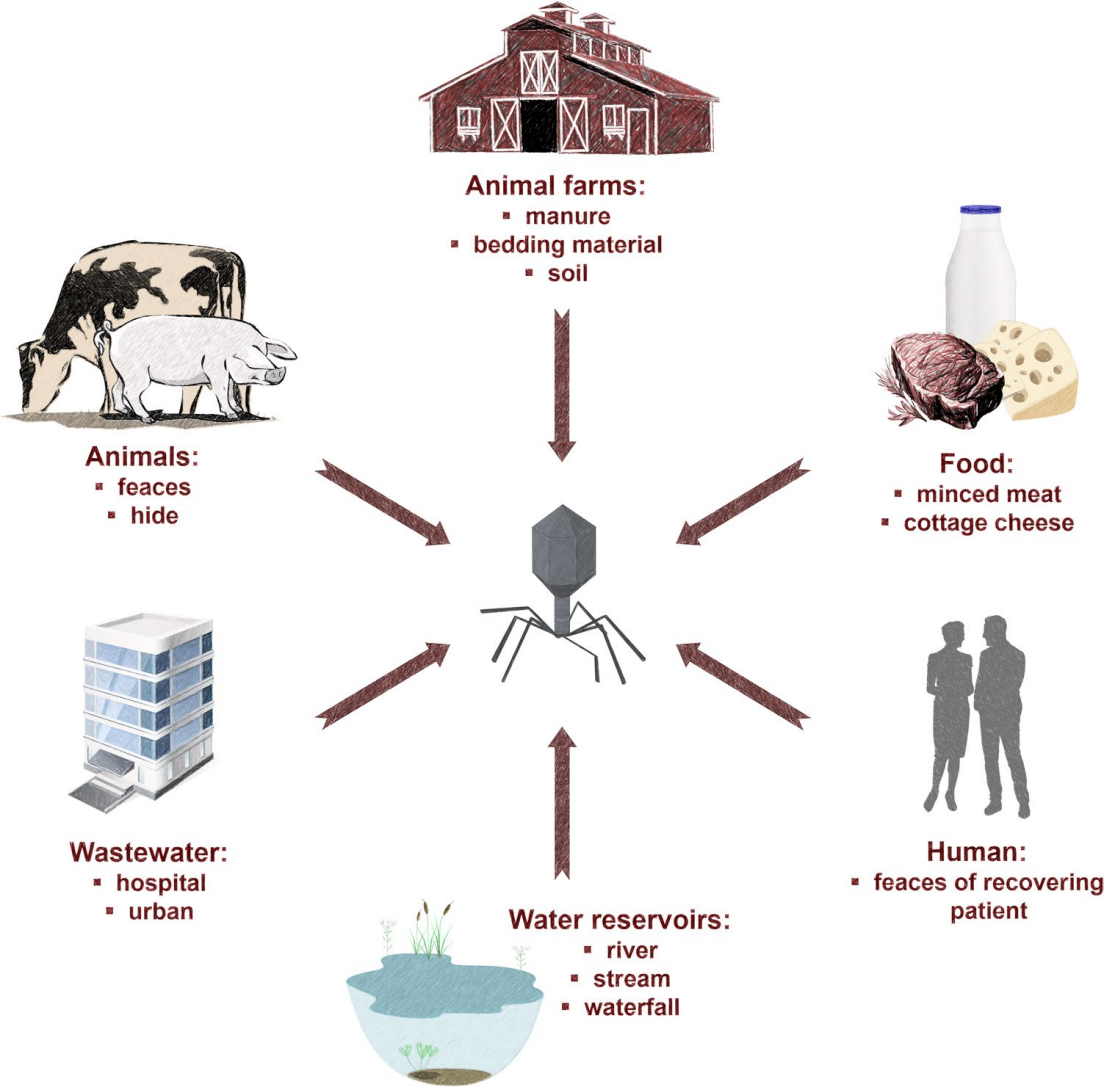
Main sources used for isolation of phages infecting EHEC

As is always the case with predators, phage occurrence is likely to coincide with the main habitats of their prey, that is, the target bacteria (Węgrzyn 2022). Therefore, phages can multiply rapidly in the presence of their host and they can be easily isolated in the host environment (Kaczorowska et al. 2019; Mangieri et al. 2020). Similarly, phages against enterohemorrhagic *E. coli* O157:H7 (EHEC) can be found in faecal samples from human patients and cattle (O’Flynn et al. 2004), especially from patients who are recovering or who have just passed an infection (Fernández et al. 2019). Given the count of human pathogenic bacteria in infected patients, hospital wastewater is a good source of phages that infect these pathogens (Latz et al. 2016). Bacteriophages infecting EHEC are also commonly isolated from different types of samples especially animal faeces (Fig. 1) (Kudva et al. 1999; Dini and De Urraza 2010; Viazis et al. 2011; Niu et al. 2012; Lee and Park 2015; Hudson et al. 2015; Amarillas et al. 2016a; Snyder et al. 2016; Zhang et al. 2017; Litt et al. 2018; Sváb et al. 2018; Akindolire et al. 2019; Kaczorowska et al. 2019; Pham-Khanh et al. 2019; Necel et al. 2020; Duc et al. 2020; Mangieri et al. 2020; Vengarai Jagannathan et al. 2021).

However, isolation procedures can be more technically demanding, especially if host bacteria are difficult to cultivate or have developed mechanisms decreasing their susceptibility to phages (Loc-Carrillo and Abedon 2011).

There are several variations of EHEC bacteriophage isolation procedures, which all follow a similar pattern. General principles are that an environmental sample that is known to contain or have frequent contact with the desired host is collected from the field. This sample could be in the form of water, soil, faecal material, sewage effluent, etc. In some cases, the phage titer will be high enough to allow direct isolation of the virus after filtration of the sample (Kropinski et al. 2012; Fernández et al. 2019). This may easily be accomplished by low speed centrifugation (e.g. 2000 × g, 10 min) followed by sterile filtration through a membrane filter, which will retain most cellular organisms but allow the passage of phage (Gill and Hyman 2010; Kaczorowska et al. 2019). Then the presence of phages is tested by visualization of plaques on plates containing the host, using the double-overlay agar method. In this technique, the host bacteria are mixed with phage filtrate and molten soft agar (Lee et al. 2012; Manohar et al. 2018; Fernández et al. 2019; Duc et al. 2020).

However, direct isolation of the virus is not always possible and enrichment steps are often required to detect the presence of the anti-EHEC phages (Sheng et al. 2006; Manohar et al. 2018; Fernández et al. 2019; Yıldirim et al. 2021). In many cases, environmental samples may be enriched for the phages of interest by cultivation in the presence of one or more of the desired bacterial hosts. Such enrichment allow to propagate of an initially small population of the desired phages until they reach a concentration easily detectable by routine plating methods. Enrichment may be also conducted by adding a filtered liquid environmental sample to a rapidly growing, early log-phase host culture and then incubation for several hours or even days, depending on the growth rate of the host. Alternatively, the raw, unsterilized sample may be added to the host culture, or the host culture along with concentrated media may be diluted in a larger volume of the environmental sample (Kudva et al. 1999; Gill and Hyman 2010; Viazis et al. 2011; Duc et al. 2020; Yıldirim et al. 2021).

In this review, we listed over 130 bacteriophages infecting EHEC bacteria that have been found and characterized in the literature (Table 1). Most of these phages are myoviruses (42%) with long contractile tail and approximate, icosahedral head, siphoviruses (1%) with long non-contractile tail and hexagonal head (Amarillas et al. 2016a; Yıldirim et al. 2021) and podoviruses (10%), characterized by short tails and icosahedral head (Dini and De Urraza 2010; Manohar et al. 2018; Akindolire et al. 2019). However, only two phages: EP75 and FEC14 are members of *Ackermannviridae* (Van Mierlo et al. 2019; Fan et al. 2021)**,** and one: the P-9 phage belongs to the *Tectiviridae* family with an icosahedral capsid and no tail (Litt et al. 2018).

**Table 1:** List of anti-EHEC phages reported in literature

No	Phage name	Source of isolation	Classification	Method of isolation	Morphology of virions and plaques	Host range	Stability of virions at different conditions	Lytic cycle	Cytotoxic effect	Food protection	Bioinformatic analysis	Proteomic analysis	References
1	PhiG17	cattle faeces	P	-	±	±	-	-	-	-	+	-	(Akindolire et al. 2019)
2	vB_EcoM-P12	sewage	M	+	+	+	+	+	-	-	+	+	(Yıldırım et al. 2018; Yıldirim et al. 2021)
3	vB_EcoM-P13	sewage	M	+	+	+	+	+	-	-	+	+	
4	vB_EcoM-P23	wastewater	M	+	+	+	+	+	-	-	+	+	
5	vB_EcoS-P24	wastewater	S	+	+	+	+	+	-	-	+	+	
6	vB_EcoM-P34	slaughterhouse	M	+	+	+	+	+	-	-	+	+	
7	C203	cottage cheese	M	+	+	+	+	+	-	+	+	-	(Sváb et al. 2018)
8	P206	poultry liver	M	+	+	+	+	+	-	+	+	-	
9	SH6	water	S	-	+	+	+	+	-	-	+	+	(Hamdi et al. 2017)
10	SH7	water	M	-	+	+	+	+	-	-	+	+	
11	KIT03	the soil of poultry farm	M	+	±	+	-	+	-	-	+	-	(Pham-Khanh et al. 2019)
12	BECP10	sewage	S	+	+	+	+	±	-	+	+	-	(Park and Park 2021)
13	P-8 O121	faeces and water from a cattle farm	M	+	+	+	+	+	-	-	-	-	(Litt et al. 2018)
14	P-10 O26	faeces and water from a cattle farm	M	+	+	+	+	+	-	-	-	-	
15	P-13 O26	faeces and water from a cattle farm	M	+	+	+	+	+	-	-	-	-	
16	P-18 O103	faeces and water from a cattle farm	M	+	+	+	+	+	-	-	-	-	
17	P-19 O103	faeces and water from a cattle farm	M	+	+	+	+	+	-	-	-	-	
18	P-21 O103	faeces and water from a cattle farm	M	+	+	+	+	+	-	-	-	-	
19	P-22 O103	faeces and water from a cattle farm	M	+	+	+	+	+	-	-	-	-	
20	J-1 O145	faeces and water from a cattle farm	M	+	+	+	+	+	-	-	-	-	
21	J-30 O145	faeces and water from a cattle farm	M	+	+	+	+	+	-	-	-	-	
22	P-11 O26	faeces and water from a cattle farm	S	+	+	+	+	+	-	-	-	-	
23	P-12 O26	faeces and water from a cattle farm	S	+	+	+	+	+	-	-	-	-	
24	P-14	faeces and water from a cattle farm	S	+	+	+	+	+	-	-	-	-	
25	P-17 O111	faeces and water from a cattle farm	S	+	+	+	+	+	-	-	-	-	
26	P-20 O103	faeces and water from a cattle farm	S	+	+	+	+	+	-	-	-	-	
27	P-9 O45	faeces and water from a cattle farm	T	+	+	+	+	+	-	-	-	-	
28	phiC119	horse faeces	S	+	+	+	-	+	-	-	+	±	(Amarillas et al. 2016a)
29	AHP24	cattle faeces	S	-	+	+	-	-	-	-	-	+	(Niu et al. 2012, 2014)
30	AHS2	cattle faeces	S	-	+	+	-	-	-	-	-	+	
31	AHP42	cattle faeces	S	-	+	+	-	-	-	-	-	+	
32	AKS96	cattle faeces	S	-	+	+	-	-	-	-	-	+	
33	FEC14	sewage	A	+	+	+	-	-	-	-	+	-	(Fan et al. 2021)
34	P-1	water	M	+	+	+	+	+	-	-	-	-	(Litt and Jaroni 2017)
35	P-2	water	M	+	+	+	+	+	-	-	-	-	
36	P-3	water	S	+	+	+	+	+	-	-	-	-	
37	P-4	water	S	+	+	+	+	+	-	-	-	-	
38	P-5	water	M	+	+	+	+	+	-	-	-	-	
39	P-6	water	S	+	+	+	+	+	-	-	-	-	
40	P-7	water	S	+	+	+	+	+	-	-	-	-	
41	O157-IOV-4	river water	M	+	+	+	-	-	-	-	-	-	(Zhang et al. 2017)
42	C14 s	calf faeces	M	+	±	+	-	±	-	+	-	-	(Vengarai Jagannathan et al. 2020, 2021)
43	V9	calf faeces	M	+	±	+	-	±	-	+	-	-	
44	L1	calf faeces	S	+	±	+	-	±	-	+	-	-	
45	LL15	calf faeces	S	+	±	+	-	±	-	+	-	-	
46	BPECO 19	sewage	P	+	+	+	±	+	-	+	-	-	(Lee and Park 2015; Seo et al. 2016)
47	EP75	sewage	A	+	-	-	-	-	-	+	+	-	(Van Mierlo et al. 2019; Shebs et al. 2020)
48	EP335	sewage	P	+	-	-	-	-	-	+	+	-	
49	e11/2	bovine slurry	M	+	+	+	+	+	-	+	-	-	(Morita et al. 2002; O’Flynn et al. 2004; Rivas et al. 2010; Coffey et al. 2011)
50	e4/1c	bovine slurry	S	+	+	+	+	+	-	+	-	-	
51	PP01	swine stool	M	+	+	+	-	+	-	+	-	-	
52	CB O103	minced meat	M	+	+	+	+	-	±	-	±	-	(Dini and De Urraza 2010; Dini et al. 2016)
53	CB O113	minced meat	M	+	+	+	+	-	±	-	±	-	
54	CC O103	minced meat	M	+	+	+	+	-	±	-	±	-	
55	CC O113	minced meat	M	+	+	+	+	-	±	-	±	-	
56	CB 60P	minced meat	M	+	+	+	+	-	±	-	±	-	
57	CB 60H	minced meat	P	+	+	+	+	-	±	-	±	-	
58	CA 933P	minced meat	P	+	+	+	+	-	±	-	±	-	
59	CA 911	minced meat	M	+	+	+	+	-	±	-	±	-	
60	CA 933D	minced meat	P	+	+	+	+	-	±	-	±	-	
61	CA 933N	minced meat	P	+	+	+	+	-	±	-	±	-	
62	MFA 45D	feaces	P	+	+	+	+	-	±	-	±	-	
63	MFA 933H	feaces	P	+	+	+	+	-	±	-	±	-	
64	MFA 60D	feaces	P	+	+	+	+	-	±	-	±	-	
65	MFA 60N	feaces	P	+	+	+	+	-	±	-	±	-	
66	MFA 45N	feaces	P	+	+	+	+	-	±	-	±	-	
67	MFA 933P	feaces	P	+	+	+	+	-	±	-	±	-	
68	ECML-4	fresh and salt water	M	-	+	+	-	±	-	+	-	-	(Abuladze et al. 2008; Sharma et al. 2009; Ferguson et al. 2013; Necel et al. 2020)
69	ECML-117	fresh and salt water	M	-	+	+	-	±	-	+	+	-	
70	ECML-134	fresh and salt water	M	-	+	+	-	±	-	+	-	-	
71	vB_Eco4M-7	sewage	M	+	+	+	+	+	+	+	+	+	(Jurczak-Kurek et al. 2016; Necel et al. 2020, 2021, 2022)
72	PE37	bovine intestine	M	+	+	+	+	+	-	+	+	-	(Son et al. 2018)
73	vB_EcoM_FFH_2	wastewater treatment plants	M	+	+	±	-	+	-	+	+	-	(Hong et al. 2014a, b)
74	vB_EcoS_FFH_1	wastewater treatment plants	S	+	+	±	-	+	-	+	+	-	
75	vB_EcoS_FFH_3	wastewater treatment plants	S	+	+	±	-	+	-	+	+	-	
76	FAHEc1	sewage	M	+	+	+	+	-	-	+	+	-	(Hudson et al. 2013, 2015)
77	AKFV33	steers faeces	S	+	+	+	+	+	-	-	+	+	(Niu et al. 2012, 2020, 2021)
78	phiE142	faeces	M	-	+	+	-	-	-	-	+	-	(Amarillas et al. 2016b)
79	phiC119	horse faeces	S	+	+	+	-	+	+	-	+	-	(Amarillas et al. 2016a)
80	PS5	raw chicken	M	+	+	+	±	+	-	+	+	-	(Duc et al. 2020)
81	OSY-SP	sewage and manure	M	+	+	+	±	-	-	+	±	-	(Snyder et al. 2016)
82	38	faeces or manure	-	+	-	+	-	±	-	-	±	-	(Viazis et al. 2011)
83	39	faeces or manure	-	+	-	+	-	±	-	-	±	-	
84	41	faeces or manure	-	+	-	+	-	±	-	-	±	-	
85	AR1	faeces or manure	-	+	-	+	-	±	-	-	±	-	
86	42	faeces or manure	-	+	-	+	-	±	-	-	±	-	
87	CEV2	faeces or manure	-	+	-	+	-	±	-	-	±	-	
88	ECB7	faeces or manure	-	+	-	+	-	±	-	-	±	-	
89	ECA1	faeces or manure	-	+	-	+	-	±	-	-	±	-	
90	HY01	swine faeces	M	+	+	+	+	+	-	+	+	+	(Lee et al. 2016)
91	FM10	farms faeces, sewage, bedding material	-	+	-	+	-	±	-	+	±	-	(Mangieri et al. 2020)
92	DP16	farms faeces, sewage, bedding material	-	+	-	+	-	±	-	+	±	-	
93	DP19	farms faeces, sewage, bedding material	-	+	-	+	-	±	-	+	±	-	
94	MS1 026	sewage	-	+	-	+	-	±	-	+	-	-	(Shebs et al. 2020)
95	MS1 045	sewage	-	+	-	+	-	±	-	+	-	-	
96	MS1 103	sewage	-	+	-	+	-	±	-	+	-	-	
97	MS1 111	sewage	-	+	-	+	-	±	-	+	-	-	
98	MS1 121	sewage	-	+	-	+	-	±	-	+	-	-	
99	MS1 145	sewage	-	+	-	+	-	±	-	+	-	-	
100	MS1 157	sewage	-	+	-	+	-	±	-	+	-	-	
101	CBA120	feedlot	-	+	±	+	+	-	-	-	+	-	(Kutter et al. 2011)
102	vB_EcoS_Rogue	-	S	-	+	+	-	-	-	-	+	+	(Kropinski et al. 2012)
103	myPSH1131	Ganges water samples	P	+	±	+	+	+	+	-	+	-	(Manohar et al. 2018)
104	rV5	faeces	M	-	±	+	-	+	-	-	+	+	(Rozema et al. 2009; Niu et al. 2009; Kropinski et al. 2013)
105	wV7	-	M	-	±	+	-	+	-	-	-	-	
106	wV11	-	M	-	±	+	-	+	-	-	-	-	
107	wV8	-	M	-	±	+	-	+	-	-	-	-	
108	SP15	bovine, sine, chicken stool	-	+	-	-	±	+	+	-	-	-	(Morita et al. 2002; Tanji et al. 2004)
109	SP21	bovine, sine, chicken stool	-	+	-	-	±	+	+	-	-	-	
110	SP22	bovine, sine, chicken stool	-	+	-	-	±	+	+	-	-	-	
111	JK16	Cork city stream	S	+	±	+	-	-	-	-	+	+	(Kaczorowska et al. 2019)
112	JK23	Connemara National Park stream	M	+	±	+	-	-	-	-	+	-	
113	JK25	Glencar waterfall	-	+	±	+	-	-	-	-	-	-	
114	JK27	Glencar waterfall	-	+	±	+	-	-	-	-	-	-	
115	JK28	Glencar waterfall	-	+	±	+	-	-	-	-	-	-	
116	JK29	Glencar waterfall	-	+	±	+	-	-	-	-	-	-	
117	JK32	Glencar waterfall	M	+	±	+	-	-	-	-	+	-	
118	JK33	Glencar waterfall	-	+	±	+	-	-	-	-	-	-	
119	JK35	sewage	-	+	±	+	-	-	-	-	-	-	
120	JK36	sewage	M	+	±	+	-	-	-	-	+	-	
121	JK38	sewage	M	+	±	+	-	-	-	-	+	-	
122	JK40	sewage	-	+	±	+	-	-	-	-	-	-	
123	JK42	sewage	M	+	±	+	-	-	-	-	+	-	
124	JK45	sewage	M	+	±	+	-	-	-	-	+	-	
125	JK55	sewage	-	+	±	+	-	-	-	-	+	-	
126	JK56	sewage	-	+	±	+	-	-	-	-	-	-	
127	JK58	sewage	-	+	±	+	-	-	-	-	-	-	
128	CM1	chicken meat	M	+	±	+	-	-	-	-	+	-	
129	CM8	chicken meat	M	+	±	+	-	-	-	-	+	-	
130	KH1	bovine faeces	-	+	±	+	-	-	+	-	-	-	(Kudva et al. 1999; Sheng et al. 2006)
131	SH1	sewage	-	+	±	+	-	-	+	-	-	-	
132	PAH6	sewage	-	+	±	+	-	-	-	-	-	-	(Alam et al. 2011)
133	P2BH2	sewage	-	+	±	+	-	-	-	-	-	-	
134	BI-EHEC	beef intestine	-	+	-	±	-	±	-	+	+	-	(Lukman et al. 2020; Dewanggana et al. 2021; L.A and Waturangi 2023)
135	BL-EHEC	beef lung	-	+	-	±	-	±	-	±	-	-	(Lukman et al. 2020)
136	Ec_MI-02	pigeon nest	M	-	+	+	+	+	-	-	+	-	(Sultan-Alolama et al. 2023)
137	EHEC-S4	soil	-	-	-	±	-	-	-	+	-	-	(Artawinata et al. 2023)
138	UFV-AREG1	stable wastewater	M	-	±	+	-	±	-	+	+	-	(Lopez et al. 2016, 2018, 2023)
139	CAM-21	lagoon slurry of a dairy farm	M	-	±	+	-	+	-	-	+	-	(Choo et al. 2023)

The table contains the categories of information provided in the analyzed publications. Horizontal lines group phages according to references. Capital letters indicate the phage historical classification based on morphology (P, M and S) or current taxonomic affiliation (A and T): *P *podovirus, *M *myovirus, *S *siphovirus, *A* *Ackermanniviridae*, *T* *Tectiviridae*. Symbols indicate the level of completeness of the provided information: ( +) complete information, ( ±) partial information, (-) no information

## Host range of phages infecting* E. coli* O157:H7

Specificity of bacteriophages to bacterial hosts is one of the features that should be considered when projecting treatment. Depending on the purpose for which bacteriophage will be used, they can be divided into specific- or wide-range of phages. For the treatment of organisms that have a natural flora needed for keeping their properties, it is better to use bacteriophage specific to the bacteria strain or serotype. However, in the treatment of multi-infections, it is better to use a virus that can infect many types of bacteria. *Escherichia coli* bacteria are one of the elements of the physiological bacterial flora of the human colon and other warm-blooded animals. Based on the above, specific bacteriophages should be used for the treatment of living animals or food infected with pathogenic *E. coli*. On the other side, if the treated object is endangered by infection by different species of pathogenic bacteria, phages with a wide range could be more useful. Moreover, to reduce the possibility of phage-resistance development in a treated host, it is recommended to use a cocktail of bacteriophages that can infect the same strains of bacteria but use different receptors for adsorption to the cell surface. Therefore, it is important to describe the range of hosts sensitive to the treatment with the analysed bacteriophages.

In the study performed by Yıldırım et al. (2018), researchers described the host range for 37 phages that were isolated using the *E. coli* O157:H7 bacterial serotypes. To check the specificity relative to the host, bacteriophages were tested against 5 different *E. coli* O157:H7 strains, 4 *E. coli* strains, 6 *S. Typhimurium*, 6 *S. enteritidis* serovars, 8 *Salmonella enterica* serovars, and also against individual representatives of 7 different Gram-negative and Gram-positive species that were *Listeria monocytogenes*, *Staphylococcus aureus*, *Bacillus cereus*, *Yersinia enterocolitica*, *Citrobacter freundii*, *Enterobacter aerogenes,* and *Enterococcus faecalis*. From 37 tested viruses, 18 bacteriophages were able to form plaques only on O157:H7 serotypes and 15 on other *E. coli* strains. What is interesting is that 8 phages could also lyse the *Salmonella enterica* serovars. In another paper, the host range of 2 rV5-like coliphages C203 and P206 isolated using *E. coli* K-12 derivative strain MG1655 was examined. Based on the efficiency of plating, both phages were able to infect tested *E. coli* O157:H7 strains, all the analysed EPEC, EIEC, and APEC serotypes, 2 from 3 multidrug-resistant *E. coli* and 3 from 6 *Shigella* strains (Sváb et al. 2018). For two phages with *Shigella flexneri* as a host, SH6 and SH7, the host range was also described. Phage SH7 was able to infect all *E. coli* K12 and *E. coli* B derivatives, all from 16 strains of *E. coli* O157:H7, 1 strain of *Salmonella paratyphi,* and *Shigella dysenteriae*. When the SH6 phage was tested, activity against 9 strains was observed. Apart from the host bacteria *S. flexneri*, the SH6 phage also infected *E. coli* K12, and *E. coli* B derivatives. When lysate of this phage was spotted on the layers with *E. coli* O157:H7, the lysis zone was observed only at high MOI without clear plaques, suggesting that the observed effect was more a result of lysis from without than phage activity (Hamdi et al. 2017). Phage phiC119 analysed by the same researchers was able to lyse 75.75% of tested *E. coli* O157:H7, while from *Salmonella* sp. only 11% of strains were sensitive (Amarillas et al. 2016a). Litt et al. (2018) analysed 19 from 45 phages, isolated from faeces using non-O157 STEC strains. The host range of phages was determined against 21 non-O157 STEC isolates with different serotypes. Bacteriophages isolated on O26 or O103 *E. coli* were not able to lyse other serotypes. Moreover, when the morphology of phages O26 and O103 indicated their belonging to myoviruses and *Tectiviridae*, respectively, the authors suggested that different mechanisms of infection of host cells can be applied for controlling various STEC populations. Niu et al. (2014) isolated 4 phages from the faeces of feedlot cattle that were able to lyse STEC O157:H7 strain R508. All phages were able to infect 100% of tested STEC O157:H7 strains but did not show any activity against all non-O157 *E. coli* strains. In another report, seven phages isolated from water and faecal samples collected from cattle showed lytic activity against 89% of the total 55 *E. coli* O157:H7 tested isolates (Litt and Jaroni 2017). Dini and De Urraza (2010) isolated 20 bacteriophages from minced meat, pork sausage, and faecal used for the enrichment of four O157:H7 strains, four non-O157 clinical isolates with serotypes O111:NM, O113:H21, O103:H2, O145:H25, and one foodborne isolate of serotype O26:H11. The host range of those phages, except CB60P, CA911, and CA933P, was the same and only differed in efficacy. Phage CA933P was the only O157-phage capable of infecting serotype O13:H6 and *S. flexneri* serotypes 2 and 3. Abuladze et al. (2008) decided to test the host range of components of the commercially used ECP-100 phage cocktail. That mixture was able to lyse 90% (100 strains) of 111 *E. coli* O157:H7. When phages present in the cocktail were tested alone, ECML-4, ECML-117, and ECML-134 could infect 70% (78), 87% (97), and 65% (72) of the analysed strains, respectively. Moreover, ECML-4 and ECML-117 could lyse only one strain from 76 non-O157:H7 strains, while ECML-134 was able to infect 24% (18 strains). The other 20 tested bacterial species were not sensitive to the treatment with a single phage or their mixture. Son et al. (2018) also tested the lytic activity of phage PE37 against 140 strains belonging to *E. coli* and *Salmonella*. PE37 was capable of lysing 100% of STEC O157:H7 and STEC O26 serotypes. It could also infect 64% of ESBLEC isolates from 10 different O-antigen groups. However, none of the tested *Salmonella* spp. was sensitive to phage PE37. In the study by Duc et al. (2020), 8 *Salmonella* phages and 10 *E. coli* O157:H7 phages were isolated from chicken products. The widest range was detected for phage PS5, which lysed 50 (67%) of 75 *Salmonella* and *E. coli* strains tested, while the narrow range was observed for phage PS7, which could infect only 7% (5/75). It is worth mentioning that phage PS5 was capable of lysing 100% (36/36) of *E. coli* O157:H7 and 41% (9/22) of *Salmonella* strains. Mangieri et al. (2020) tested 20 bacteriophages isolated from different sources on 31 STEC strains. The phage isolated from bovine faeces showed the widest range of host bacteria by infection of all the 31 STEC strains. Additionally, 11 other phages (55%) could lyse more than 70% of the used strains. Isolated from feedlot phage CBA120 appeared to be highly specific for *E. coli* O157:H7, infecting only one of the 72 ECOR collection strains and 13 out of 17 tested O157:H7 strains. Moreover, CBA120 did not plate on any of the 107 tested *E. coli* strains isolated from cows. Determination of the host range by spot test showed that myPSH1131 phage was able to lyse 31 of the 53 *E. coli* isolates that belonged to EPEC, EHEC, ETEC, EAEC, UPEC, and one strain of unknown pathotype (Manohar et al. 2018). Niu et al. (2009) evaluated the host range of four phages (rV5, wV7, wV8, and wV11) against* E. coli* O157:H7 isolates originating from cattle and humans. Phages wV7, rV5, wV11, and wV8 were able to lyse 422, 342, 321, and 407 of the total 422 STEC O157:H7 isolates, respectively. Interestingly, phage wV7 infected all of the 297 bovine and 125 human isolates. Moreover, all of the used bacteria were susceptible to at least one bacteriophage. PP01 phage (Morita et al. 2002) isolated from the faecal sample also showed specificity to serotype O157:H7 of *E. coli* bacteria, with an inability to lyse other tested *E. coli* or non-*E*. *coli* strains. Interestingly, researchers identified potential receptor on bacterial cell for this bacteriophage. They proved that the host bacterial mutant resistant to reinfection with PP01 had lost the major outer membrane protein OmpC. Complementation by *ompC* from an O157:H7 strain but not from *E. coli* K-12 resulted in the restoration of PP01 susceptibility, suggesting that the OmpC protein serves as the PP01 receptor. This knowledge might help design the composition of a phage cocktail. While most of the host-range experiments focus on using many pathogenic bacteria, Necel et al. (2020) decided to test the susceptibility of pathogenic, laboratory, and commensal *E. coli* bacteria for infection with phage vB_Eco4M-7. Tested phage was able to form plaques on lawns of 16 *E. coli* O157:H7 STEC strains, 3 non-STEC *E. coli* O157:H7, 11 *E. coli* O157 STEC, and 4 *E. coli* O157 non-STEC strains. Phage vB_Eco4M-7 could not infect *E. coli* O26 STEC isolates from stool or 41 other *E. coli* non-O157 strains, including non-pathogenic *E. coli* bacteria or any tested *S. flexneri*, *S. enterica*, *Bacillus* sp., *P. aeruginosa*, *E. faecalis*, *S. aureus*, *Klebsiella* sp., and *Acinetobacter* sp. strains.

As shown above, many bacteriophages able to infect *E. coli* O157:H7 bacteria have a wide host range and sometimes also the additional ability to lyse other bacterial strains from *Escherichia*, *Shigella,* and *Salmonella* genus, especially when isolation was performed using non- *E. coli* strains. It is worth mentioning that there are also speculations that bacteriophages with a wider host range are more often isolated from poor in nutrients environments containing small amounts of bacteria, while more specific viruses might be found in bacteria-rich habitats (Khan Mirzaei and Nilsson 2015). However, most of the phages presenting a wide host range interact with many strains of pathogenic bacteria, which implicates the potential of bacteriophages to be used as biocontrol agents or as therapy factors for infected animals.

## Phage resistance to external factors

Bacteriophages can be more labile compounds than other antimicrobials, which can make them unstable outside a specific range of environmental conditions (temperature, pH, salt concentrations) (Fernández et al. 2019). Any deviation from the optimal conditions can lead to inactivation of the viruses (Jończyk et al. 2011). Thus, bacteriophages used in therapy should have a wide stability range. Phage virions must be able to maintain activity during the production, storage, and administration stages. Several intrinsic and extrinsic factors of environments can affect phage stability, which in turn might decrease the efficacy of phage therapy. These factors vary greatly depending on the specific phage (Jończyk et al. 2011).

The first important factor affecting phage stability is temperature. The available literature indicates that phages infecting EHEC show high thermal stability. An example are Eco-phages like vB_EcoM-P13, vB_EcoM-P12, vB_EcoM-P23, vB_EcoS-P24, and vB_EcoM-P34, which retained their infective activity under heat treatment applied at 50–60℃ for 5–60 min (Yıldırım et al. 2018; Yıldirim et al. 2021). BECP10 phage was very stable at 25–45 °C (Yıldirim et al. 2021). Litt et al. (2018) have isolated P-11 and P-12 phages targeting O26 STEC, P-8 phage targeting O121 STEC, and P16 phage targeting O45 and O111 STEC, which remained viable for 60 min at 70 °C. Interestingly, O26 and O121 STEC–infecting phages remained viable even at 90 °C. Additionally, all these phages remained stable under refrigerated (4 °C) and frozen (-20 °C and -80 °C) storage. Similarly, phages isolated by Duc et al. (2020) were viable in the temperature range (40–60 °C) and in cold storage. Also, phage vB_Eco_4M-7 was resistant to low (-20 °C) and high temperatures (95 °C) (Jurczak-Kurek et al. 2016).

These properties make them potentially interesting in the context of biotechnological applications, e.g., as biocontrol agents in the food industry (Jurczak-Kurek et al. 2016). As an example, low temperatures (2–8 °C) are commonly used during the distribution and storage of many foods. High temperatures, on the other hand, will allow the phages to survive the heat treatments. Whereas, in phage therapy, the viral particles have to remain infective from the moment of administration to the patient until they reach the target pathogen, so they have to survive e.g. a higher body temperature, as bacterial infections usually cause fever (Topka et al. 2019; Fernández et al. 2019).

Another important factor is the stability of bacteriophages under a wide pH range. This is especially important for oral application. Phages must be able to resist the highly acidic gastric passage to enter the intestine at a high enough concentration to be effective and to enable infection and lysis of pathogens (Dini and De Urraza 2010). Thanks to the stability in the wide pH range, phages could be used as biocontrol agents in the food or food-animal industry. The acidic or alkaline environment of the animal gastrointestinal tract could affect the viability of phages during application. An example are bacteriophages P-1, P-2, P-3, P-4, P-5, P-6, and P-7 recovered by Litt and Jaroni (Litt and Jaroni 2017) from beef cattle environment targeting *Escherichia coli* O157:H7. These viral particles remained stable for 24 h at a wide pH range (1–11). Similar stability was observed for phages PS5 (Duc et al. 2020), vB_Eco_4M-7 (Jurczak-Kurek et al. 2016), and Ec_MI-02 (Sultan-Alolama et al. 2023).

However, most phages are acid-sensitive and cannot tolerate acidic conditions of the stomach. For this purpose, anti-acid agents are used, which may increase phage survival (Sabouri et al. 2017). For example, the phage cocktail used by Tanji et al. (2005) was not stable at acidic pH, so CaCO_3_ was used to protect phage virions. What is important, the gastrointestinal tract of animals is an anaerobic environment where EHEC, facultatively anaerobic microorganisms, can survive and grow (Sabouri et al. 2017; Jubelin et al. 2018). However, it has been reported that activity in anaerobic conditions has been analysed for only a few phages. An example is a cocktail of phages EP16, PP17, and SP22 which was able to significantly decrease the *E. coli* O157:H7 number in such conditions (Kunisaki and Tanji 2010).

It has been reported that the form of storage of the phage particles affects their stability. Some phages remain active for a longer time when stored in a liquid-like growth medium. However, another possibility is storage of the phage particles in a dry powder, which can be produced by e.g., lyophilization (Fernández et al. 2019). The advantages of having lyophilized phages are that they can withstand long-term storage, present an increased half-life and can be used in different pharmaceutical formulations (Merabishvili et al. 2013). Studies presented by Manohar et al. (2018) showed that the use of sucrose and the combination of sucrose plus gelatin can restore the viability of freeze-dried *Escherichia* phage myPSH1131 during long-term maintenance at 4 °C. Another example is the lyophilized phage, CA933P, which was also able to significantly reduce EHEC adhesion to Hep-2 cells. This phage is combined with a microbial mixture of probiotic microorganisms to combine the lytic effect of phage and the ability of microorganisms to reduce the pathogenicity and the cytotoxic effect of EHEC on epithelial cells in vitro (Dini et al. 2016). The lyophilized phages infecting EHEC were found to be good candidates for therapeutic purposes (Manohar et al. 2018; Fernández et al. 2019). Potential candidates for phage therapy and/or food protection should also be highly resistant to various disinfectants and chemicals. For example, vB_Eco4M-7 and ECML-117 phages showed complete resistance to 10% soap and dish soap, Line-Antibacterial 70, Virusolve, SDS, and chloroform (Necel et al. 2020). In turn, phage PS5 was relatively stable in NaCl applied at a concentration from 1 to 11%, suggesting that this phage may have a long shelf life in a food environment (Duc et al. 2020).

## Lytic properties of phages against EHEC bacteria

**Fig. 2:**
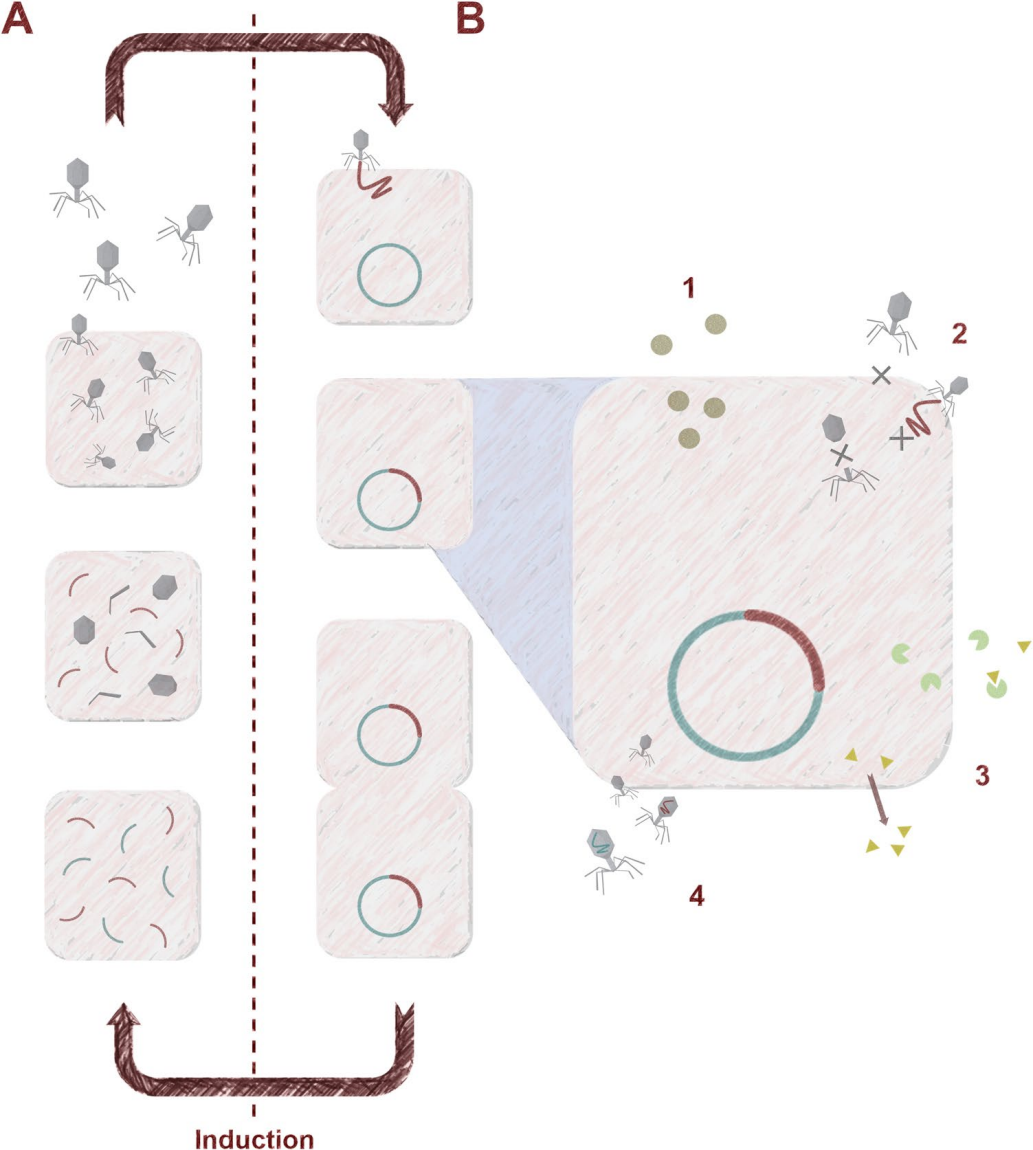
Diagram of the phage development cycles: lytic (**A**) and lysogenic (**B**). Numbers indicate different possible effects of prophage presence: 1—Production of virulence factors like toxins and biofilm inducers; 2—Superinfection resistance through receptor blocking, abortive infection and assembly disruption; 3—Resistance to antimicrobials due to the production of inactivating enzymes and efflux pumps promotion; 4 – Horizontal gene transfer

Bacteriophages are the viruses of bacteria that can be divided into two groups based on their life cycle, virulent and temperate (lysogenic) (Fig. 2). When temperate phages are infecting bacterial cells, they can choose a lytic life cycle in which phage progeny is produced and bacterial cells are ultimately killed or a lysogenic cycle where the genome of bacteriophage is integrated into the genome of bacteria in the form of a prophage. However, there are many reasons why temperate phages should be avoided in therapy (Gill and Hyman 2010; Hassan et al. 2021). Therefore, it is recommended to use only virulent phages, which can replicate only through the lytic life cycle.

To characterize lytic development, some experiments like the efficacy of adsorption, lysis profile, and one-step growth experiment are performed. All of those procedures bring information about the time of lysis, the most efficient multiplicity of infection, and the count of the phage progeny (burst size). The short latent time under 15 min (time between adsorption and initiation of progeny production) together with the large number of progeny particles (> 50 PFU/cell) indicate high infectivity and lytic activity against the host.

Adsorption rate of vB_EcoM-P12, vB_EcoM-P13, vB_EcoM-P23, vB_EcoS-P24, and vB_EcoM-P34 phages after five minutes of incubation with host bacteria was very high and ranged between 92.7–97.5%. The latent period of vB_EcoS-P24 was 10 min, while for other phages it was 15 min. Analysed burst size showed that tested bacteriophages can produce 98 to 144 new phage particles from a single cell. Moreover, it was observed that the optimum MOI, in which the most number of phage particles is produced in one cycle, ranged between 0,001 and 0,1 (Yıldirim et al. 2021). In the one-step growth experiment with phages C203 and P206 infecting *E. coli* O157:H7 Sakai strain, the latent period was 60 min, with the burst size of 1000 (Sváb et al. 2018). In another study (Hamdi et al. 2017), phages SH6 and SH7 had a 16- and 23-min long latent period. Interestingly, while infection with SH6 phage resulted in about 103 progeny virions, the burst size of phage SH7 was only 26 on average. However, this experiment was performed on *S. flexneri* SF1 host strain, which may not reflect development in EHEC bacteria. Pham-Khanh et al. (2019) decided to test the efficacy of propagation of KIT03 phage in its initial host (*E. coli* NBRC 3972), *E. coli* O157:H7, and *S. choleraesuis* and obtained an approximate burst size of 39, 51, and 37 phage particles per cell, respectively. Interestingly, the tested phage was more efficient at a higher MOI (1 or 10), but at a lower MOI (0,1) was able to delay bacterial culture re-growth. Litt et al. (2018) characterized the lytic cycle of 19 phages able to lyse non-O157 STEC. Phages belonging to the myoviruses, siphoviruses*,* and *Tectiviridae* exhibited a latent period between 8–37, 10–21, and 30 min, respectively. Moreover, the phages showed a wide range of burst sizes, from 12 to 794 phage particles per cell. In 2017, Litt and Jaroni (2017) published a paper in which phages recovered from beef cattle were examined. The adsorption rate of these phages was low, with only 41% of adsorbed phages after 20 min. The maximum percent of adsorbed virions was observed after 80 min for P-4 phage, but it was still only 60% of the initial phage population. According to the results obtained by Amarillas et al. (2016a), the life cycle of phiC119 takes about 60 min to complete, its latent period takes 20 min, while an average burst size counts 210 phage particles per cell, as noted after 55 min since adding the phage. Lopez et al. (2023) showed that phage UFV-AREG1 has a life cycle lasting 50 min with a 25 min latent period, and the burst size was about 18 PFU per cell.

Observed characteristics of lytic cycles and their single stages showed that most of the isolated phages indicate strong lytic activity, which strengthens their possible role in the regulation of the EHEC population both in industry and healthcare.

## Toxicity of phage preparations toward eukaryotic cells

It is common knowledge that phages cannot infect eukaryotic cells. Bacteriophages bind to specific receptors on bacterial cells, which are absent in eukaryotic cells. As such, phages are generally considered to be harmless to humans and animals (Domingo-Calap and Delgado-Martínez 2018; Endersen and Coffey 2020). The safety of the bacteriophages is also supported by the fact that they are extremely common in the environment (Vinícius Pimentel Rodrigues et al. 2021). They have been isolated among others from water, food, soil, or even the human body (Barr 2017). In human organisms, bacteriophages are found wherever bacteria exist: on the skin, oral cavity, lungs, intestines, or urinary tract (Batinovic et al. 2019). Constant exposure to phages in the environment and no visible side effects may indicate the safe usage of phage preparations.

To demonstrate the safety of bacterial viruses, interactions between bacteriophages and eukaryotic cells or organisms are investigated. Manohar et al. (2018) used the *Galleria mellonella* larvae model for assessing the in vivo activity and toxicity of *Escherichia* bacteriophage myPSH1131. *G. mellonella* is a useful model organism, increasingly being used in scientific research. Bacteriophage myPSH1131 has a broad host range, that showed lytic activity against 31 isolates, including EHEC. Researchers have observed 100% larval survivability after injection of phage lysate, which indicates that there were no toxic byproducts in the lysate. Moreover, they demonstrated effective bactericidal effects in reducing *E. coli* populations. This phage showed 100% effectiveness when three doses were given at 6 h intervals. Necel et al. (2020) investigated the cytotoxicity of vB_Eco4M-7 phage particles to mammalian cells. They have assessed the viability of mouse embryonic fibroblasts (BALB/c3T3) after treatment with this phage. Used phage showed no cytotoxic effect on mammalian cells and no decrease in viability of these cells was observed after 24 h incubation with phage lysate. However, when the effects of vB_Eco4M-7 on *E. coli* O157:H7 (ST2-8624) co-cultivated with these mammalian cells were tested, results demonstrated that phage treatment has not significantly reduced the cytotoxic effect of *E. coli* O157:H7 bacteria on eukaryotic cells.

Currently, there are only a few research that assessed the cytotoxic effect of phages infecting EHEC bacteria. Nevertheless, by analyzing the results obtained for bacteriophages capable of lysing other species of bacteria, we can observe interactions between phages, bacteria, and cells. Shan et al. (2018) showed that bacteriophage phiCDHS1 is more virulent to bacteria mixed with human cells. In this case, phage phiCDHS1 reduced *Clostridium difficile* cell number more effectively in the presence of HT-29 cells than in in vitro studies, in which alone bacteria were used. Research also showed that the phage phiCDHS1 was not toxic to HT-29 cells, and phage-mediated bacterial lysis did not cause toxin release and cytotoxic effects. In subsequent studies, no cytotoxic effect of *Acinetobacter baumannii* phage BS46 on mouse fibroblast 3T3 cells was detected after 24 h exposure using different assays (Henein et al. 2016).

Importantly, all information presented above may suggest that bacteriophages were in general not toxic to eukaryotic cells. However, when testing the cytotoxicity effect, it is important to examine the contamination of phage lysates with bacterial toxins, which is one of the phage therapy safety concerns (Doub 2021; Chung et al. 2023). The presence of e.g. endotoxins, may not only disturb the results of experiments but also modulate a therapy effect through i.a. interactions with immune response elements (Clarke et al. 2020; Chung et al. 2023). Therefore, a lot of methods of phage purification from bacterial debris were created. Most of them, are based on ultracentrifugation, ultrafiltration, or chromatography, and each one shows remarkable efficiency in the clearance of bacterial contaminants from phage lysates, increasing their safety (Luong et al. 2020; Rebula et al. 2023; Roshankhah et al. 2023).

## The use of phages in food protection

Contamination of food products with pathogenic bacteria, like enterohemorrhagic *E. coli*, is one of the major concerns of the food industry, as it may lead to large economic losses through the costs of, e.g., healthcare, utilization of contaminated food, recall from the sale, and payment of compensations. Only in the U.S., the estimated cost of food safety incidents is around $55,5 billion per year (Scharff 2015). The outbreak of EHEC in Germany in 2011, caused by serotype O104:H4, besides the high economic loss, led to over 4,000 hospitalizations with 54 fatal cases (Köckerling et al. 2017). It is also worth mentioning that outbreaks of EHEC are reported every year, which enforces looking for a strategy to minimize the risk of food contact with these pathogenic bacteria (Kanayama et al. 2015).

**Fig. 3:**
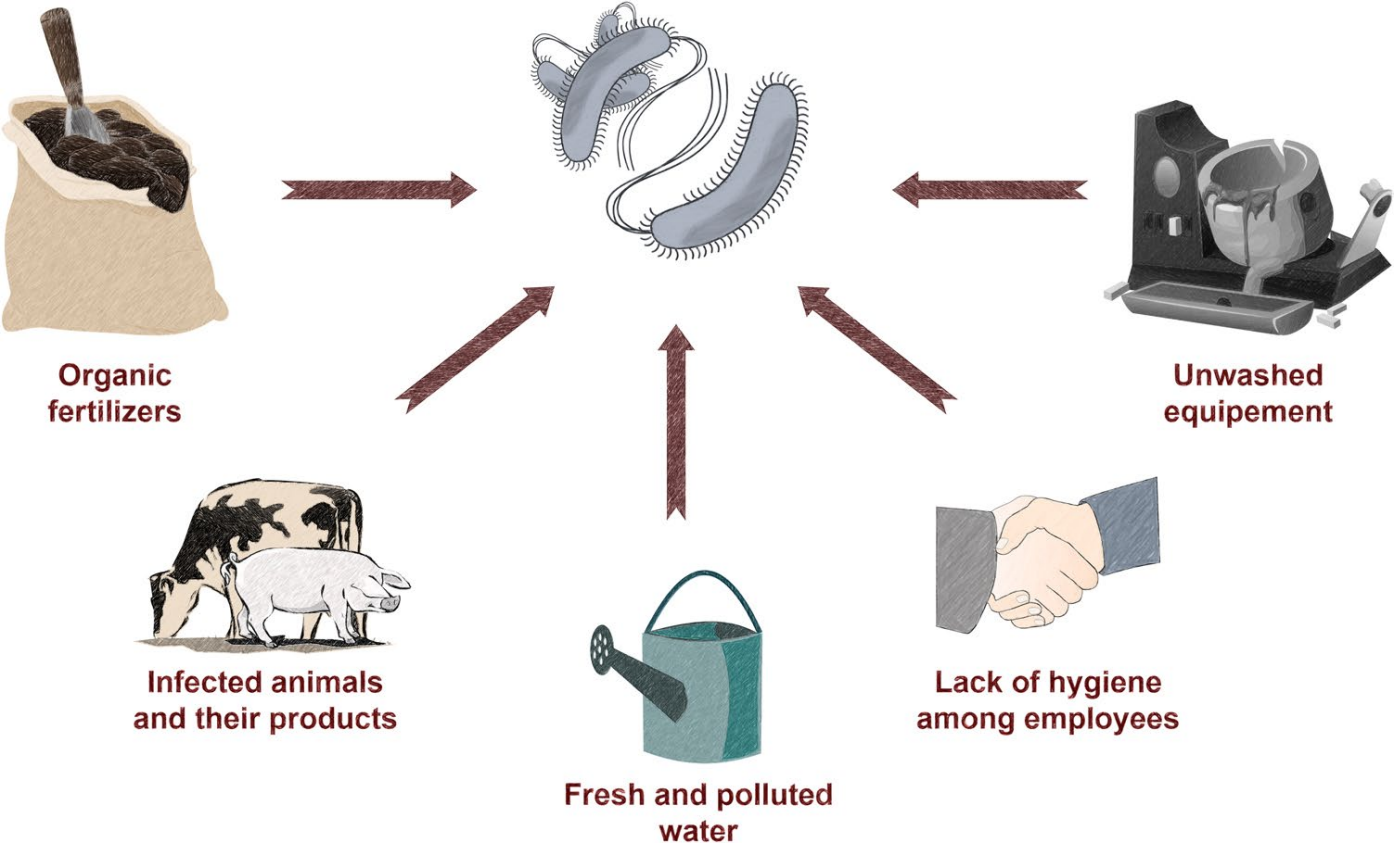
Main risk factors of food contamination with EHEC in industry

The main problem with food contamination is that it may occur in every step of food processing, from the pre-harvesting period to storage (Fig. 3) (Galié et al. 2018). Chemical sanitization procedures may not only enhance bacteria survival through the development of insensitivity to them, but also cause damage to food and alter the environment (Abuladze et al. 2008; Stanford et al. 2021). Moreover, many STEC strains are characterized by a wide range of resistance to antibiotics, which are usually used to treat bacterial infection (Al-Marri et al. 2021). Zhao et al. (2001) analysed 50 isolates of STEC, where 78% of the isolates showed resistance to two or more antibiotics from different classes. The above problems create the need to find a factor that works at all stages of food protection.

Bacteriophages, as the natural enemies of bacteria, became one of the potential food protectants. At first, bacteriophages are usually specific to host bacteria and cause the decrease of only pathogenic bacteria without disruption of the natural flora. Moreover, the usage of these viruses does not damage the food or change its quality. It is also worth mentioning that bacteriophages can penetrate the biofilm matrix and gain access to bacterial cells. Based on the food manufacturing procedures, food production may be divided into 4 periods: pre-harvest, harvesting, food processing, and storage. Bacteriophages can be applied in all of them, and as shown in the literature, they provide efficient effects on EHEC bacteria (Sheng et al. 2006; Callaway et al. 2008; Sharma et al. 2009; Rivas et al. 2010; Boyacioglu et al. 2013; Ferguson et al. 2013; Hudson et al. 2015; Liu et al. 2015; Malik et al. 2017; Sváb et al. 2018; Son et al. 2018; Stratakos and Grant 2018; Abdelsattar et al. 2019; Vikram et al. 2020; Yin et al. 2021).

### Bacteriophages in a direct animal treatment

In the first period of food production, the main source of pathogenic bacteria is direct contact with infected animals, hydration of animals/crops with infected water, or usage of organic fertilizers. Since the main reservoirs of STEC are animals (especially cattle), some research focuses on using bacteriophages directly on animals, which also may reduce the amount of EHEC released to the environment and stop the further spreading of these bacteria. Sheng et al. (2006), showed that using phage SH-1 alone or in combination with KH-1 was able to reduce the number of bacteria in mice to a non-detectable level in 2 to 6 days, while in the control group, bacteria were detected after more than 10 days. Based on the above results, researchers decided to treat experimentally infected steers by application of SH1 and KH1 phages directly to the rectoanal junction mucosa (RAJ) at a phage/bacterium ratio over 100 and by using water with 10^6^ phage particles per ml (PFU/ml) for hydration through the whole experiment. The average number of *E. coli* O157:H7 bacteria in rectoanal mucosal swab samples declined sharply to the level of 2.4 logs after 1 day, compared to 3.9 logs in the control group. After 7 days of the treatment, the number of bacteria, cultured from the RAJ, in the phage-treated group decreased further to an average of 1.4 logs, while the control group was estimated at 2.7 logs. However, pathogenic bacteria were still detected in most samples at the end of the experiment. Other results were obtained by Callaway et al. (2008). For oral treatment of sheep, inoculated with 10^10^ cells of *E. coli* O157:H7 strain 933, a cocktail of 8 phages isolated from feedlot cattle faeces was used. After 72 h from cocktail administration, the bacterial population was significantly reduced in the phage-treated group compared to the controls at 72 h and maintained its downward trend. Moreover, this treatment reduced gut populations of *E. coli* O157:H7 in the rumen, cecum, and rectum. Phage particles were detected after treatment in caecal and rectal samples, but in ruminal samples, they were found only in 2 of 10 sheep. Additionally, the more effective decrease was obtained when phages/bacteria were at a 1:1 ratio rather than 10:1 or 100:1. In another report, phages e11/2 and e4/1c were examined as biocontrol agents of the *E. coli* O157:H7 population using an ex vivo rumen model and then compared to the results of an in vivo experiment on cattle (Rivas et al. 2010). In the ex vivo experiment, phages were used separately, and both phages were able to significantly reduce the number of cells. However, phage e11/2 decreased the CFU to the non-detectable level at direct plating after 1 h of treatment, while when phage e4/1c was used, bacteria were still detected, and their number remained unchanged after a decrease observed at 2 h post-administration. In both trials of the experiment with cattle, a cocktail of phages was used. In the first trial, where around 16-month-old cattle were inoculated with 10^10^ bacterial cells, oral application of the cocktail showed no effect on the number of *E. coli* O157:H7 in faeces. Cell count decreased in both the control and phage-treated groups after 48 h and was still detected after 7 days following enrichment. The number of phage particles in faeces also didn’t differ between samples taken from the analysed groups on the indicated days. In the second trial, where 8–9-year-old cattle were infected with the same number of cells as in the first trial, rumen and faecal samples were analysed. The number of bacteria decreased to a similar level in both the control and cocktail-treated groups after 92 h, but in faecal samples, the number of cells was significantly lower than those from the rumen samples. Moreover, while in rumen samples phage particles were decreasing after dosing, in faecal samples phage plaques were not observed, even following enrichment. As the authors suggested, no effect of bacteriophages on the bacteria population might be due to lack of exposure of phages to bacteria, low concentration of phage particles, inactivation by physiological conditions, or binding within the digestive tract. Some researchers propose using encapsulation of phages in oral treatment, to increase the number of phage particles surviving the digestive system and arriving at the target place (Malik et al. 2017; Abdelsattar et al. 2019). In in vitro experiments, it was shown that microencapsulated phages are more resistant to simulated gastric fluids and bile acids. When tested in vivo, orally administered encapsulated phages were more effective in decreasing the number of *E. coli* O157:H7 in rats than free, unprotected phage particles (Yin et al. 2021).

Some studies have shown that animal hides are the most crucial source of pathogenic bacteria (Mather et al. 2007; Stromberg et al. 2016). Treatment of infected cattle hide with earlier mentioned bacteriophages e11/2 and e4/1c showed no effect when samples were immediately taken after treatment. However, after 1 h of incubation, it has shown a more effective bacteria reduction ability of phages than water washing. The authors suggested that for commercial applications, a longer time should be used, and because of the processing line speed, it is not possible to obtain it during slaughtering. Therefore, phages should be applied in the pre-harvest period (Coffey et al. 2011). In another study, the significant effect of bacteriophages on the reduction of bacterial population in hide was observed for 3 out of 5 strains of EHEC. Host bacteria were treated with phages that in in vitro experiments showed the strongest lytic activity, which highlights that infection capacity obtained in those types of experiments may not be observed during industrial usage. Moreover, the used MOI or EOP was not crucial for effectiveness, as reduction of *E. coli* O111 was not detected, despite having the highest in vitro EOP and applied MOI (Tolen et al. 2018). In a study performed by Arthur et al. (2017), which used commercialized bacteriophages to spray cattle before processing, no significant reduction of *E. coli* O157:H7 was observed on hides. The authors indicated that one of the reasons for the lack of efficacy might be due to the large amount of organic matter and frequent contamination by other pathogens in the cattle environment.

### Animal products protection with phages

Since the main reservoir of EHEC is animals, especially cattle, meat, and animal products are one of the major sources of those bacteria (Ferens and Hovde 2011). The first outbreak of the previously mentioned *E. coli* O157:H7 strain was in 1982 in the USA, where infections were caused by consumption of undercooked burgers. At that time, this strain was rare, but it quickly dominated the EHEC population in the country (Pacheco and Sperandio 2012). Therefore, several studies are showing the positive effect of bacteriophages on the reduction of EHEC populations in food, especially serotype O157:H7. Hudson et al. (2013), experimentally infected raw and cooked beef with *E. coli* O157:H7 and checked the reduction effect of bacteriophage FAHEc1 after 24 h at 2 temperatures: 5 °C and 24 °C. While in most of the samples treated at 24 °C re-growth of bacteria was observed, in samples with a low concentration of bacteria (< 1 log 10 CFU/cm^2^) and phage concentration at 3.4 × 10^5^ PFU/cm^2^, bacteria did not re-growth. However, as the authors noted, keeping meat for 24 h at that temperature represents gross temperature abuse. Treatment of both kinds of meat with phage at 5 °C, which is a more representative temperature in food processing, decreased the number of bacteria to the level that remained stable for 24 h (Hudson et al. 2015). In another paper, the activity of the commercially available bacteriophage cocktail EcoShield™ (Intralytix, USA) against *E. coli* O157 was analysed at 4 °C and 12 °C for 7 days. The cocktail was spread onto the infected beef surface to achieve an MOI of 100. In samples stored at 12 °C, the number of bacteria significantly decreased after one day. However, on the third day, the population started to re-grow but was still significantly lower than the phage-free sample, where bacteria were constantly growing. At a lower temperature of 4 °C, the cocktail was able to reduce the number of bacteria, despite the inhibition of bacteria multiplication that was observed in the control group (Stratakos and Grant 2018). A similar effect was obtained earlier for infected ground beef stored at 10 °C for 24 h, where the pathogenic bacteria population was reduced by about 95%. Vikram et al. (2020) examined the efficacy of the EcoShield PX cocktail for reducing the levels of *E. coli* O157:H7 in different types of food inoculated with bacteria at 3.0 log CFU/g. Usage of 2 higher concentrations of phages (10^6^ and 10^7^) resulted in significant reductions of up to 97% in all types of food. Application of phages to treat a lower concentration of bacterial cells (1 to 10 CFU/10 g) on beef chuck roast reduced the prevalence of EHEC in meat samples by more than 80%. Liu et al. (2015) used 4 bacteriophages to control the population of *E. coli* O157:H7 on beef at 4 °C, 22 °C, and 37 °C temperatures. In all cases, the bacteriophages significantly reduced the number of bacterial cells and maintained the bacterial count reduction trend. While samples at 22 °C and 37 °C were only analysed for 6 and 3 h, respectively, the reductional impact of treatment at 4 °C was still observed after 144 h. Another phage, PE37, used on artificially contaminated with STEC O157:H7 raw beef, was able to reduce the counts of bacteria after 24 h of incubation at 25 °C and 8 °C by 2,3 and 0,9 log CFU/piece, respectively. Moreover, when phage was applied on raw beef contaminated with STEC and ESBLEC strains, the number of viable cells also significantly decreased (Son et al. 2018). Sváb et al. (2018) decided to test the reductional potential of phage P206 on minced meat. Again, phage was able to reduce the population of pathogenic bacteria on meat by 10^4^ and 10^2^ when MOI of 0,6 and 0,06 were used, respectively. Similar results, to those previously described, were obtained by other researchers on different kinds of meat (Park et al. 2012; Seo et al. 2016; Shebs-Maurine et al. 2020). However, any differences between incubation times and the temperature turned out to be a significant factor of phage activity, which was also observed in different studies (Artawinata et al. 2023; L.A and Waturangi 2023).

Some studies also describe the effect of bacteriophages on milk and dairy, which might be contaminated during the milking process or processing at the facility. It has been shown that a cocktail of 3 phages: EC6, EC9, and EC11 completely inhibited *E. coli* ATCC 25922 and *E. coli* O127:H6 in UHT and raw milk at 25 °C and under refrigeration temperatures (5–9 °C) (Tomat et al. 2013). A number of these cells also decreased in non-treated raw milk, but the phage cocktail was able to eliminate these strains more rapidly. Another decreasing effect on the STEC population was observed where phages DT1, DT6, or a cocktail of them were used in the process of milk fermentation. O157:H7 STEC strains were rapidly and completely inactivated by both phages and cocktails. Moreover, bacteriophages did not influence the number of *Streptococcus thermophilus* used as a lactic starter in the fermentation process.

### Phage usage in the plant products care

Currently, a diet consisting largely of plant products is recommended for maintaining health. Therefore, raw vegetables and fruits have become more often the reason for food poisoning in recent days. The risk factors for microbial contamination of them include, *i.e.,* growing on clay-type soil, the application of contaminated manure or water, and wild animals (Park et al. 2012; Alegbeleye et al. 2018). Sharma et al. (2009), tested the ECP-100 phage cocktail (currently named EcoShield™, Intralytix) on fresh-cut cantaloupes and green-leafy lettuce. Phage treatment of cantaloupes inoculated with *E. coli* O157:H7 B6914 gfp 86 showed a reduction of bacteria number on days 2, 5, and 7 when incubated at 4 °C, with the largest difference at day 2 (2.87 log CFU/ml). However, when stored at 20 °C, a significant difference between phage-treated samples and control was observed only on day 5. When bacteriophages were sprayed on fresh-cut lettuce, the count of viable bacteria decreased shortly after application on day 0. Importantly, phages were sprayed on lettuce, while on cantaloupes cocktail was spotted, which might be the reason for the different effectivity on day 0. Moreover, by adding the centrifugation step, researchers tested if the observed reduction was caused by the activity of phages attached to the bacteria on the surface of the lettuce or by the action of free phage particles left in homogenates during sample preparation. After the removal of free phage particles, the bacterial count was still decreased similarly to the previous experiment without this step. Ferguson et al. (2013) tested if the earlier mentioned cocktail can protect fresh-cut lettuce from cross-contamination with *E. coli* O157:H7 by immersion of lettuce in a highly concentrated suspension of phage cocktail for 2 min before bacteria inoculation. Results were compared not only to control but also confronted with samples where phages were applied after pathogen inoculation and followed exposure to chlorine for 30 s. Immersion of lettuce for 2 min in suspension with 9.8 log PFU/ml followed by storage at 4 °C significantly reduced the number of bacteria on the 1st and 3rd days of the experiment and decreased it to the non-detectable level on day 7. Interestingly, it turned out that spraying of inoculated earlier lettuce was more efficient in decreasing the bacterial population on day 0, when compared to the control. Moreover, spraying was more effective in combination with chlorine treatment, than with the cocktail alone. In summary, both methods showed the reducing effect of the cocktail, but spraying was more effective in the immediate decrease of bacterial population. In another study, the same cocktail was sprayed on spinach, romaine, and green-leaf lettuce, followed by incubation at 4 °C and 10 °C to mimic storage conditions (Boyacioglu et al. 2013). Additionally, it was tested if there were changes in the effectiveness of phages when used in an aerobic or modified atmosphere (5% O_2_, 35% CO_2_, 60% N_2_). Under aerobic conditions, the phage mixture was able to reduce the number of *E. coli* cells on spinach, green leaf, and romaine lettuce by 1.19, 3.21, and 3.25 logs of CFU/cm^2^, respectively. However, when samples were stored at 4 °C under an altered atmosphere with high CO_2_, the decrease in cell number was higher when compared to samples stored in aerobic conditions. The same tendency was observed in samples stored at 10 °C. Magnone et al. (2013) tested the influence of EcoShield™ on *E. coli* O157:H7 that was artificially applied to strawberries, cantaloupes, and broccoli. In this study samples were treated with a phage cocktail before storage, the levulinic acid after storage, a combination of both agents, or the chlorine alone. Moreover, experiments were performed using solutions prepared with potable tap water or challenged water (water with 2.5 g/litre of organic carbon and dissolved solids). In samples from the tap water group, the applied phage cocktail did not significantly reduce the bacterial count on all surfaces. Importantly, in the challenge water group, the reduction of bacterial viability was observed in cantaloupe and broccoli when compared to the control. However, the most effective treatment, manifested with the highest decrease in bacterial count in all samples, was obtained when a combination of a phage cocktail and a produce wash was used. Additionally, the wash with 200-ppm free available chlorine in challenge water experiments was the least effective method. Results suggest that the application of phages during a standard produce wash may be an effective way to fight *E. coli* O157:H7 even in the presence of higher organic content. In another paper, phage *E. coli* phage OSY-SP isolated from manure was tested on green pepper infected with *E. coli* O157:H7 EDL933 or baby spinach inoculated with fluorescently labeled *E. coli* O157:H7 B6–914 (Snyder et al. 2016). What is important is that pepper samples were treated earlier with UV radiation to eliminate the natural microbiota, while baby spinach was used without prior decontamination to avoid damage. Therefore, baby spinach was inoculated with the marker-containing strain. Samples were rinsed in phage solution for 2 or 5 min and incubated at 4 °C, 25 °C, or in combination of both temperatures. In this experiment, researchers decided to engage 2 types of control. First, fresh produce was only inoculated with bacteria, and second, inoculated vegetables were rinsed with medium to see if the observed decrease came from mechanical or phage action. Rinsing of contaminated baby spinach leaves indicated that the activity of phage caused 1,6 log reduction in the number of bacteria. In the case of cut green pepper stored at 4 °C, phage activity was estimated as 1,6 log, while in samples incubated at 25 °C temperature for the first 4 h followed by storing at 4 °C for the remaining 68 h, the count of bacteria was reduced by 2,2 logs. However, the difference between storage at 4 °C and temperature combination is not significant. In another study, vB_Eco4M-7 phage, which may infect many *E. coli* O157:H7 strains, was used as a biocontrol agent of bacterial growth in cucumber (Necel et al. 2021). For this purpose, researchers used sliced cucumber, not the whole vegetable, to mimic conditions for severe infections. Treatment of the cucumber slices with bacteriophages for 6 h resulted in a significant decrease of the inoculated *E. coli* bacteria at all tested MOIs, ranging from 0,0001 to 10. After 24 h of incubation, bacterial re-growth was observed. However, changes in the bacterial count were still significant when compared to the control. What is interesting, is that the most efficient reduction was noted when a lower MOI was used (0,0001–0,01).

In most of the discussed experiments applying higher temperature, up to 25 °C, the reduction of the bacterial count was spectacular in the first hours of the experiment, but after longer storage, the bacterial re-grow was observed. Importantly, at 4 °C which is a more suitable temperature to simulate storage conditions in the food industry, phage-treated samples obtained a more efficient decrease in the cell number during longer storage. The above papers perfectly describe the potential of phage treatment of contaminated vegetables and fruits. Moreover, studies have shown that when designing procedures using phage preparations, the storage temperature, infection rate, and method of application should be considered.

## Conclusions

Nowadays, in the MDR era, it is extremely important to search for another method of bacteria eradication, especially the one focused on prevention. Lack of influence on food natural flora if phage specific to host bacteria is used, together with results described in literature indicate the remarkable effect of bacteriophages on the populations of EHEC bacteria in industry. However, for further studies concerning phage application in food industry, it is important to focus on the isolation of new phage strains and to perform their detailed morphological, genetic, and physiological characterization. One of the most crucial features that have to be analysed is the type of phage life cycle. Bacteriophages, considered to be used in phage therapy, should always be lytic since lysogenic phages increase the risk of, e.g., the transfer of virulence genes to other bacteria. Moreover, phage products should be created taking into account the influence of other ingredients on the stability of virions, and storage conditions, but also including procedures that minimize the risk of the presence of bacterial residues, endotoxins, and products encoded by the antimicrobial resistance genes.

Additionally, scientists are currently discussing several other concerns. One of them is the risk of phage resistance development. However, various studies show that resistant mutants often result in lower virulence or antibiotic sensitivity and that the arms race between bacteria and phages promotes coevolution, helping phages to overcome resistance (Capparelli et al. 2010; León and Bastías 2015; Chan et al. 2016, 2018; Castledine et al. 2022; Warring et al. 2022; Wang et al. 2023). This underlines the potential of phages to be used, especially in combination with antibiotics or other antimicrobials. Another important topic is the loss of phage specificity against bacteria. Most often, the activity of phages is concentrated on strains from the same species or family, so a loss of specificity to particular strains might result in a wider range of infected strains from the same family. On the other hand, for phages infecting bacteria closely related to those belonging to natural flora, this phenomenon might be disadvantageous. In the case of EHEC, loss of specificity might threaten the natural flora present in an environment like the intestine. However, the effect of a wider host range of phages is considered to be less endangering than the usage of antibiotics, which are active against bacteria from different families from the beginning. Additionally, some methods allowing expansion of host range can be found in literature (Mapes et al. 2016; Bull et al. 2022; Ponce Benavente et al. 2024). These are pointing out the possibility of “adapted” phages to increase therapy effectiveness without the need of isolation of new phages.

For now, in most countries, there are no legal regulations allowing the common use of bacteriophages. However, a lot of products were registered and used as antimicrobials. These are representing a wide range of delivery systems that are usually based on lyophilized, encapsulated, or immobilized in different materials phages. In the food industry, phages can be used at different stages of production: directly on both plants and animals, as sanitizers to avoid the formation of bacterial biofilms, or directly on food as a preservative. For human therapy, phages can be administered orally, topically (transdermal, dental, etc.), parenterally (e.g., intramuscular or intravenous), or through airways via nebulization or inhalation (Loh et al. 2021).

For food protection, several products were developed, like ListShield™, SalmoFresh™ or EcoShield™ PX (www.intralytix.com). Besides the last one, there are also many other products aimed to fight EHEC strains, which show remarkable effects and are continuously updated to expand effectivity against newly appearing strains (De Melo et al. 2018; Huang et al. 2022). In the European Union (EU), only Phageguard Listex™, which is active against Listeria strains (https://phageguard.com), was approved to be used in food processing. In medicine, phage therapy is commonly available only in a few countries, like Georgia or Russia. In the EU it is still treated as an experimental way to fight pathogens, while in the USA there are only a few products that obtained FDA permission. These are intended to be used against pathogens like adherent-invasive *E. coli*, *Pseudomonas aeruginosa*, or *Staphylococcus aureus* (Huang et al. 2022). However, this might change in the near future because many phage-based drugs are currently analysed in clinical trials, providing promising results (Hitchcock et al. 2023; Swenson et al. 2024).

Undeniably, there is a long path to introduce phages to common usage in the food industry, however, both the direction and obstacles are now well known, implicating that it is a matter of time when phages will become the crucial antibacterial agent, also in the fight against pathogens like EHEC.
